# Scoping review of methodology for aiding generalisability and transportability of clinical prediction models

**DOI:** 10.1186/s41512-026-00237-8

**Published:** 2026-10-01

**Authors:** Kritchavat Ploddi, Glen P. Martin, Maurice O’Connell, Matthew Sperrin

**Affiliations:** 1 Division of Informatics, Imaging & Data Sciences, Faculty of Biology, Medicine and Health, University of Manchester, Manchester, UK; 2 School of Medicine, Faculty of Education & Health Sciences, University of Limerick, Limerick, Ireland

**Keywords:** Generalisability, Transportability, Clinical prediction models, Causal inference, Dataset shift

## Abstract

**Background:**

Generalisability and transportability of clinical prediction models (CPMs) refer to their ability to maintain predictive performance when applied to new populations. While CPMs may show good generalisability or transportability to a specific new population, it is rare for a CPM to be developed using methods that prioritise good generalisability or transportability. There is an emerging literature of such techniques; therefore, this scoping review aims to summarise the main methodological approaches, assumptions, advantages, disadvantages and future development of methodology aiding the generalisability and transportability of CPMs.

**Methods:**

Relevant articles were systematically searched from MEDLINE, Embase, medRxiv, arxiv databases until August 2023 using a predefined set of search terms. Extracted information included methodology description, assumptions, applied examples, advantages and disadvantages.

**Results:**

The searches found 1,761 articles; 172 were retained for full text screening; 18 were finally included. We categorised the methodologies based on whether they are data-driven or knowledge-driven, and whether they are generalisable or transportable to specific target population. Data-driven approaches range from data augmentation to ensemble methods and density ratio weighting, while knowledge-driven strategies rely on causal methodology.

**Conclusions:**

Future research could focus on comparing these methodologies across simulated and real datasets to identify their strengths and weaknesses in broad applications, as well as synthesising these approaches for enhancing their practical usefulness.

**Supplementary Information:**

The online version contains supplementary material available at https://doi.org/10.1186/s41512-026-00237-8.

## Background

**Fig. 1:**
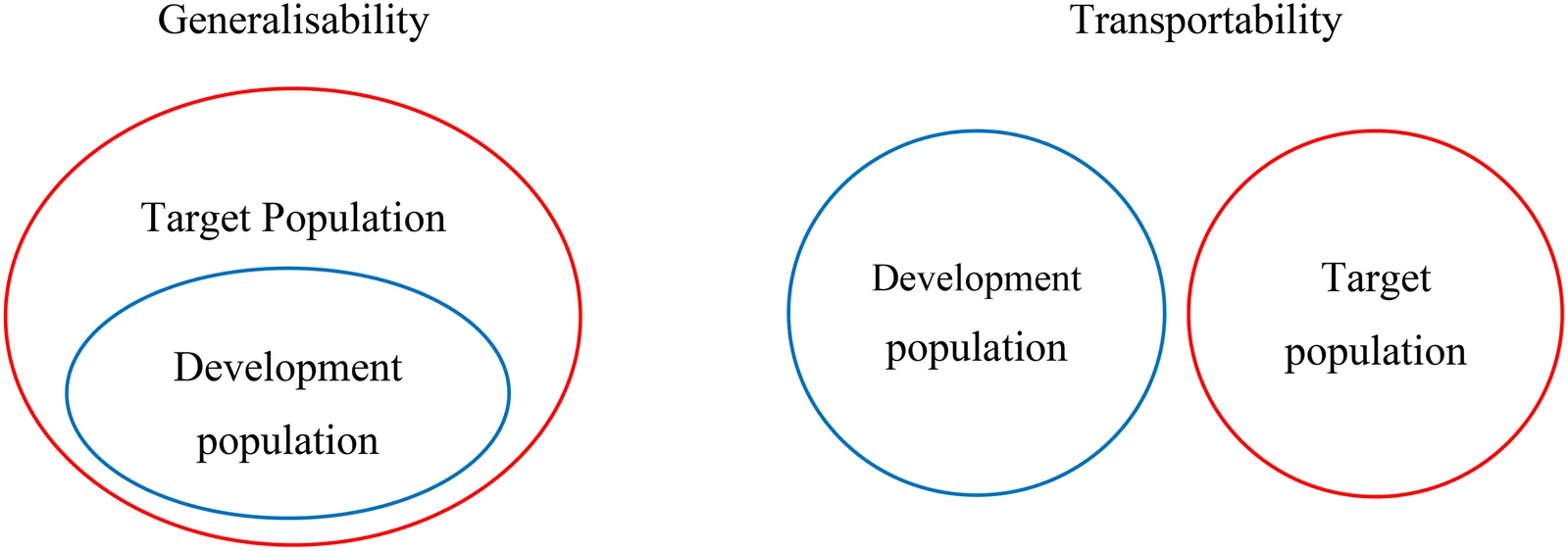
Diagram showing a relationship of the development and target populations: (left) generalisability, (right) transportability

Generalisability and transportability collectively refer to the extent to which results of research can be applied to settings and populations other than those in which the research was originally conducted. These include an application of the results to different geographical locations, times, or environments. In clinical prediction [1, 2], these terms refer to the ability of a clinical prediction model (CPM) to maintain predictive performance when applied to new settings and populations [3, 4]. Generalisability of a CPM refers to its ability to maintain predictive performance in target populations (the populations in which the model is intended to be applied) which, in general, distributionally overlap with the data the model was developed on; transportability of a CPM refers to its ability to maintain predictive performance in a target population that is separate to the data the model was developed on (Fig. 1). Both generalisability and transportability are typically assessed by conducting external validation of a CPM in the target population [5, 6]. A CPM is occasionally developed or evaluated in populations that differ from the intended target, often due to data availability or convenience [7, 8]. This is one of the reasons why it fails to transport to the target population. In addition, if the target population is small, supplementing it with data from a larger population may be a choice. This mismatch complicates the model’s application in the actual target population. CPMs are usually developed to minimise predictive error in the training data, using methods such as penalised likelihood to mitigate overfitting. This does not guarantee that the models are generalisable or transportable; that is, the predictive error may not be minimised in the target population or setting that differs from the development population. This is often attributed to changes in the distribution of data (such as predictors, outcomes, or predictor-outcome relationships) between development and target population/setting: so-called data distribution shift [9, 10]. The shift could be due to changes in technology (e.g., changes in coding definitions by different EHR platforms used in different hospitals [11]), population and/or setting (e.g., taking a model developed in hospitalised patients to a community population), or behaviour (e.g., adoption of an effective CPM may result in better treatment outcomes and this changes may gradually reduce model performance [11, 12]).

A common way in which poor generalisability and transportability of a CPM is handled is by applying updating methods to tailor the model to the new target population/setting [13, 14]. These methods include updating the regression coefficients according to the new data [15, 16], combining multiple similar models with meta-analysis techniques [4, 17, 18], and continuously updating the models [19, 20]. However, if the generalisability and transportability of a CPM could be improved during the initial development phase, it would readily work in new target populations without any adjustment, thus reducing the need for further updates and the resources required for such updates.

There is emerging literature around methods that seek to develop CPMs with improved generalisability and transportability, including using causal methods such as a selection diagram or a directed acyclic graph [21, 22]. Causal machine learning is an umbrella term for applying causal methods to prediction or machine learning models, including improving their generalisability and transportability [23]. However, these methods are rarely used in practice. One reason for this might be a lack of awareness of these methods and a current lack of a summary of them for a broad audience.

As such, the aim of this scoping review was to identify methodology that develops clinical prediction models such that they aid generalisability and/or transportability. We aim to summarise the main methodological approaches, along with reported assumptions, reported advantages and reported disadvantages. In addition, we will identify any gaps and future development required in methodology for developing generalisable and transportable CPMs.

## Methodology

### Definitions

The *development population* is the population from which the data used to develop the CPM was (randomly) sampled. The *target population* is the population in which we are interested in applying the CPM. *Generalisability* refers to maintenance of predictive performance for a target population that contains the development population as a non-random subset of an over-arching population—defined as a theoretical population whose members share the same data-generating process. Transportability is maintenance of predictive performance for a target population that is distinct (non-overlapping) from the development population, which belongs to a different over-arching population. (See Fig. 1)

For example, assume there are two UK over-arching populations, primary care and secondary care—each with a consistent data-generating process nationwide. We consider developing a CPM to predict cardiovascular disease (CVD) in adults hospitalised in Manchester using local data, and subsequently evaluating its performance across all UK hospitals. Since Manchester hospitals are a non-random subset of all UK hospitals, then this represents evaluating the model’s generalisability. In contrast, if we wish instead to explore the use of this model in predicting CVD risk in patients who visit primary care services, then this would be evaluating the transportability of the model.

The previous idea of geographical generalisability could be extended to other types: temporal generalisability—generalisability across calendar time—and generalisability across demographic groups such as ethnicity, sex, age, and level of deprivation, which collectively address prediction fairness.

### Scope of review

This scoping review included any methodology that aid generalisability and transportability during CPM development. We focused on those developed using structured data. The review did not include methodology that takes developed models and make an adjustment to improve generalisability and transportability, such as model updating techniques, for which there are existing reviews [13, 14]. We followed the preferred reporting items for systematic reviews and meta-analyses 2020 (PRISMA 2020) [24]. The PRISMA study checklist was reported in the Appendix section.

### Inclusion/exclusion criteria

#### Inclusion

- Papers proposing any method that aims to aid the generalisability or transportability of a CPM, at the time of model development.
- Applied papers reporting the development of a CPM, by using specific methods intended to aid the generalisability or transportability of said model.

#### Exclusion

- Non-English papers.
- Letters, comments, editorials, and conference abstracts with no information to allow assessment of proposed methods.
- Papers that lack sufficient information on how the method improves generalisability or transportability.
- Papers only considering an external validation of an existing CPM.
- Papers considering model updating, transfer learning or other adjustment methods to improve generalisability or transportability after the initial model has been developed.
- Papers without prediction using structured data (i.e. data in a tabular format where each column represents predictors or an outcome of interest and each row represent each observation).

### Search Strategy

Defining search keywords a priori for methodological papers can be challenging, because of inconsistent methodological terms used by different researchers without standardized terminology [25]. We therefore defined an initial set of search terms using the titles, abstracts and keywords of the following papers related to the generalisability of CPMs that we were aware of prior to conducting this review:

- Bellamy, D., Hernán, M. A., & Beam, A. (2022). A structural characterization of shortcut features for prediction. European Journal of Epidemiology, 37 [6], 563–568 [26].
- Piccininni, M., Konigorski, S., Rohmann, J. L., & Kurth, T. (2020). Directed acyclic graphs and causal thinking in clinical risk prediction modeling. BMC Medical Research Methodology, 20 [1], 179–179 [27].
- Subbaswamy, A., Schulam, P., & Saria, S. (2018). Preventing Failures Due to Dataset Shift: Learning Predictive Models That Transport [21].
- Fehr, J., Piccininni, M., Kurth, T., & Konigorski, S. (2022). A causal framework for assessing the transportability of clinical prediction models (p. 2022.03.01.22271617). medRxiv [22].
- Jong, V. M. T., Moons, K. G. M., Eijkemans, M. J. C., Riley, R. D., & Debray, T. P. A. (2021). Developing more generalizable prediction models from pooled studies and large clustered data sets. Statistics in Medicine, 40 [15], 3533–3559 [28].

Specifically, from these papers, we identified the following search terms:

- generalisab*.
- generalizab*.
- transportab*.
- “dataset shift”.

We searched for papers having titles including any of these terms (generalis* OR generaliz* OR transportab* OR “dataset shift”) to find research mainly focusing on generalisability or transportability. To further filter the results for CPMs, we used search terms proposed by Geersing et al. [29] for identifying prediction modelling studies:

1. (Validat* OR Predict*.ti. OR Rule*) OR (Predict* AND (Outcome* OR Risk* OR Model*)) OR ((History OR Variable* OR Criteria OR Scor* OR Characteristic* OR Finding* OR Factor*) AND (Predict* OR Model* OR Decision* OR Identif* OR Prognos*)) OR (Decision* AND (Model* OR Clinical* OR Logistic Models)) OR (Prognostic AND (History OR Variable* OR Criteria OR Scor* OR Characteristic* OR Finding* OR Factor* OR Model*)).
2. Stratification OR ROC Curve OR Discrimination OR Discriminate OR c-statistic OR c statistic OR Area under the curve OR AUC OR Calibration OR Indices OR Algorithm OR Multivariable.

Articles were searched based on titles containing terms related to generalisability AND terms in [1] OR [2], i.e. (generalisability terms) AND (CPM term [1] OR CPM term [2]). The aforementioned five papers were used as a validation of this final search (i.e., we ensured the above five papers were identified from our final search string).

Articles from the arXiv database were exempt from the application of the Geersing et al. criteria, as papers found using generalisability terms were relatively fewer than those from other databases and they typically include recent methodology applying in other fields and having the potential for clinical prediction. The exact search strategy was described in the Appendix section.

### Information sources

The primary author applied the above search terms to MEDLINE, Embase, medRxiv and arXiv, and performed abstract screening and subsequent full-text screening on the filtered articles according to the inclusion/exclusion criteria. We used Ovid Medical Research Platform to implement search strategies for MEDLINE, Embase and medRxiv databases. MedRxiv and arxiv are pre-print databases and are also included as many recent methodological papers are not yet published in peer-review journals. We searched these databases on 4 August 2023.

### Snowballing (Citation search)

To compliment the database search, we further applied the inclusion/exclusion criteria to papers which cite, or were cited by, the papers identified from the search (after full-text screen). In addition, we were also aware that other researchers have published titles relating to generalisability in general or other settings but not directly aiding the generalisability of a CPM, such as methods to generalise RCT results to observational data. We did not build search terms based on these but rather did citation searching based on these studies [30–33].

### Screening and selection

After articles were identified and deduplicated from the databases, the primary author (KP) performed screening in two sequential steps: title/abstract screening and full-paper screening. This was done by scanning key text sections to determine whether articles met the inclusion and exclusion criteria. The other authors provided support whenever eligibility remained inconclusive.

### Data extraction

Through full-paper reading—which involved a detailed review of each article—the primary author extracted a pre-defined set of information from the included articles. This extraction was done in a single phase in accordance with the inclusion and exclusion criteria. The other authors provided guidance whenever uncertainty arose regarding the extracted data or its interpretation. The following information was extracted:

- Title, authors, year of publication.
- Whether generalisability, transportability or both, based on the definition of the target population.
- Methodology, including the family of proposing methodology (e.g., causal inference), description of the methodology, underlying reported assumptions.
- Whether the methodology is transportable to specific target population.
- Whether domain knowledge is required when applying the methodology.
- Reported advantages, including reported key strengths of the methodology. This potentially includes sound methodology with explicit examples.
- Reported disadvantages, including reported limitations, challenges or knowledge gaps of the methodology.
- Any applied examples of the proposed methodology, including in medical and other areas.

## Results

**Fig. 2:**
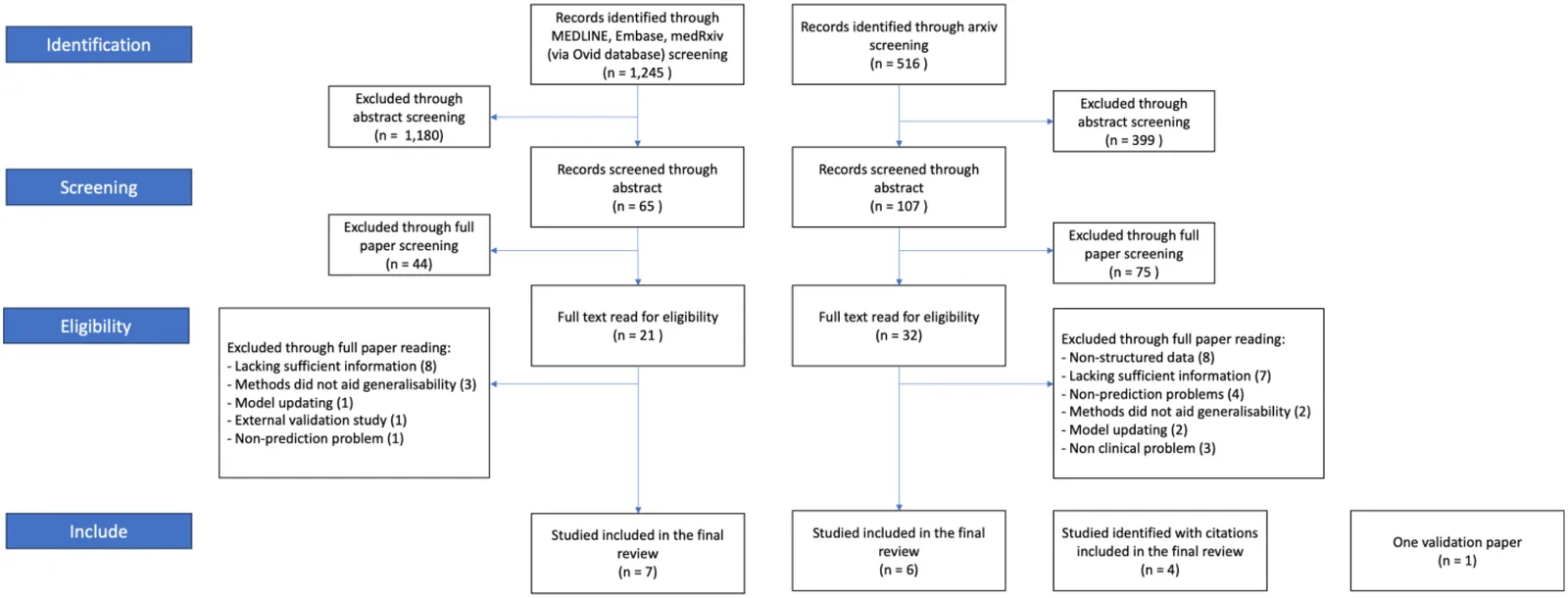
PRISMA 2020 flow diagram showing the various stages through the scoping review

The database searches identified 1,761 articles. Of these, 172 were retained for full text screening. Finally, 13 articles were eligible for inclusion into the study. Through citation searching, four additional papers were identified and included. One paper from the validation set [26] was not found by the search strategy, but nevertheless it was included in the review, giving the final 18 papers eligible for the scoping review (Fig. 2).

The scoping review has identified several methodologies to aid the generalisability and transportability of clinical prediction models. To aid in summarising, we have categorised them into: [1] data-driven methodologies that enhance the robustness of prediction models [27, 28, 34–43], and [2] knowledge-driven methodologies that integrate causal reasoning [21, 22, 26, 27, 38, 44–46], (Table 1 and Supplementary Table 1). The data-driven approach aims to enhance model robustness by adopting methodologies primarily informed by data. In contrast, knowledge-driven methods utilise existing domain knowledge about causal relationships between variables to map this understanding into mathematical equations and statistical distributions prior to the application of empirical data. There were four articles focusing on applying machine learning methods [34–36, 40], while the other 14 articles were methodological focusing on developing the new one or extending the existing methodology [21, 22, 26–28, 37–39, 41–46].

In addition, we classified methodologies based on whether they aid a CPM to be generalisable to target population or transportable to specific target population. According to Fig. 1 (left), methodologies aiding generalisability within the development population are generalisable to the target population, which is an over-arching population of the development. In contrast, methodologies targeted for a new, distinct population are transportable to that specific target population, as per Fig. 1 (right). We found methodologies that are transportable to specific target population sometimes require data from that population, including density ratio weighting and other reweighting approaches, or may not have the requirement but comes with additional assumptions (knowledge-driven and causal inference methods). Both classifications are shown in Table 1.

**Table 1:** Classification of the methodology aiding generalisability and transportability of clinical prediction models

	Generalisable to target population	Transportable to specific target population
Data-driven	1. Data augmentation [28, 41] 2. Ensemble modelling [35]	1. Density ratio/importance weighting [37] 2. Adversarial validation [43] 3. Measurement error comparison [37]
Knowledge-driven	-	1. Selection diagram [21, 45] 2. Graph surgery estimator [21] 3. Shortcut predictor [26] 4. Distributionally robust model [38, 39] 5. Minimising worst-case risk [38, 39] 6. Optimal stable and immutable sets [38, 39] 7. Markov blanket, Parent-child nodes [22, 27, 46] 8. Causality-aware prediction by counterfactual approach [44]

### Data-driven methodologies that are generalisable to target population

This group of methods involves developing a prediction model using heterogeneous data [28, 35, 41], which can be collected, for example, from multiple centres or locations across different geographical regions. Exposing the model to heterogeneous development data will dilute location- or cluster-specific patterns and this can lead to a more generalisable model [41]. Additionally, the model’s transportability could be higher if this heterogeneous development data partly represents the target population in respect to covariate and/or outcome distributions. Some of the methods also enhance the data diversity by augmenting the training data, either through analysing clustered data [28] or by embracing cohort heterogeneity (i.e. mixing data from multiple heterogeneous cohorts to dilute cohort-specific patterns) [41]. The strategies improve generalisability by capturing more general patterns of the development data and less subgroup-specific patterns.

#### Data heterogeneity

de Jong et al. extended the Internal-External Cross-Validation (IECV) technique to develop models that generalise well [28]. IECV non-randomly clusters the training data, ensuring that the selection introduces a level of heterogeneity that mirrors real-world scenarios. Stepwise selection is then performed, with the optimality criteria including a generalisability penalty, thus identifying an optimal prediction model that demonstrates best performance on unseen, heterogeneous data, which potentially results in a better generalisable model. This also can be seen as finding a parsimonious model on a heterogenous data. To illustrate, the SIECV method can be applied to develop a CVD prediction model using data from various countries. In this approach, each individual country serves as a validation set for models developed using the remaining countries, which are then aggregated into the final SIECV model using meta-analytic methods. This results in an aggregated model expected to generalise effectively without being transportable to any specific country. de Jong showed in their case studies that predictors selected by SIECV led to more stable models; however, there was variation in calibration and discrimination across populations (Supplemental Table 1, No.10). The most significant limitation of SIECV is its reliance on meta-analytic aggregation methods; these function as hyperparameters that directly influence its performance.

Similarly to de Jong et al., Reps (2022) applied ensemble techniques with the objective of improving the model’s generalisability to new populations [35]. They trained an L1-regularised logistic regression model on five distinct healthcare databases, which reflect the heterogeneity on the development populations. These base learners were then ensembled using various techniques, such as weighted and stacked ensembles. The ensembled models are learned from different base learners and different databases so that they can generalise to the new data. Additionally, the authors used a ‘leave-one-database-out’ approach, where one of the five databases was held out to validate the performance of the ensemble models trained on the remaining four [35], and all held-out validation process was repeated until all databases were evaluated. The models identified from a ‘leave-one-database-out’ are expected to have the highest performance on the held-out database, as they were specifically tuned to this database. This approach is similar to SIECV, which are used to identify models that are least overfit to the development data. However, it differs in how individual models are aggregated; SIECV uses meta-analysis, while this method employs ensemble techniques. Yet, the authors found that while the method improved discriminative performance on a new unseen database, it still had poor calibration (i.e. poor calibration-in-the-large and calibration slope) on the new database (Supplemental Table 1, No. 9). This might reflect that this approach still does not find the optimal prediction model that works best in the new unseen data for all performance metrics. Another limitation is that the original findings came from population in the USA, which may not applicable to other countries.

### Data-driven methodologies that are transportable to specific target population

These are methods that additionally take data from target population as part of development data. By exposing parts of the target population during initial model development, the models are more likely to be transportable to that specific target population. The additional data from the target population could be only covariate data or with an addition of outcome data. This process mainly aids transportability to the specific target population.

#### Density ratio weighting

Density ratio weighting involve adjusting the characteristics of individuals in the development data to resemble those in the target data in terms of predictors or covariates by assigning weights to individuals in the development population based on their similarity to those in the target population [34, 36, 40, 42]. These weights are usually represented by the density ratio, which, although calculated slightly differently across various articles, retains a similar underlying concept: making the development population more representative of the target population. The formal definitions are provided in the Appendix.

Gao et al. [34] and Steingrimsson et al. [40] indirectly estimated density ratio weights. Gao et al. fitted L1- and L2-penalised logistic regression, and random forest, on combined data from development and target populations predict the probability of each individual being sampled from the target population (Supplemental Table 1, No. 9). The fitted models give the odds of being sampled from the target population, which indirectly represent the density ratio. Note that this estimation requires covariate data from the target population but not outcome data. CPMs with weighted likelihood estimation using these odds were fitted to predict sepsis in hospitalised patients. The study demonstrated that density ratio weighting significantly improved the generalisability of sepsis detection models under covariate shift. While discrimination (AUROC) consistently improved, calibration (Brier scores) did not show uniform improvement, particularly in complex models with extensive hyperparameters (Supplemental Table 1, No. 1).

Steingrimsson et al. also estimated the density ratio as the probability of sampling from the target population divided by the probability of sampling from the development population. They termed this the inverse of the odds of being from the development population. The inverse odds is used for developing prediction models with weighted logistic regression in a manner similar to that of Gao et al. Furthermore, the inverse odds is not identifiable in non-nested designs where the development and target populations are completely different and sampling proportions from each populations are unknown unless entire observations are obtained. However, Steingrimsson et al. demonstrated that the inverse odds are proportional to the inverse odds in the training set under certain assumptions, which are used to fit the weighted logistic regression model. A more detailed description is provided in the Appendix. To illustrate, they conducted a simulation study comparing standard and weighted prediction models to evaluate their performance when transported to a new target population. They found that while the inverse-odds weighting approach provides minimal benefits for correctly specified models, it significantly reduces mean squared error (MSE) for incorrectly specified models by tailoring them to the target population’s distribution (Supplemental Table 1, No. 5).

Dockès defined importance weights, which are the ratio of the joint probability distribution of covariates and outcome in the target over the development data [36]. This is equal to the density ratio previously described. These importance weights were used to modify the training errors or loss functions. Dockès also noted that when the importance weights are applied with Bayes’ rule in cases of covariate shift exhibiting selection bias, the inverse odds similar to those proposed by the previous researchers is obtained.

The limitations of density ratio weighting are: first, that it is not applicable in the concept shift case, where the relationship between predictors and outcome vary between populations; second, it relies on access to the target population as covariate data from the target is required (Supplemental Table 1, No. 1,5).

#### Adversarial validation

Qian et al. who introduced the concept of adversarial validation to enhance the transportability of credit score prediction models [43]. This method involves building a classifier to predict the probability of being from the target population, $\:P(s=0\:|\:X)$, on the development data. The predicted probabilities are used during an internal validation to find the model that best transports to the target population by either selecting a subset of an internal validation dataset with the highest probabilities, or using the probabilities as sample weights during fitting the models. By modifying internal validation datasets toward target population, a developed model is tailored to be transportable to specific target population, hence improved transportability. Nevertheless, the application of this methodology in clinical prediction models has been limited.

#### Measurement error adjustment

Pajouheshnia et al. argued that apart from dataset shift, transportability issues could also arise from variations in measurement error patterns between development and target populations, even in the absence of dataset shift [37]. They formally demonstrated that disparate measurement error distributions across populations can lead to suboptimal model performance in the target population. The authors suggested that ensuring consistent measurement processes for predictors in both populations could enhance the transportability of a prediction model and mitigate the risk of performance degradation. This method does not directly improve generalisability and transportability per se, but rather provide the guidance to do so, i.e. accounting for differences between the development data and target population. This is similar to imputing missing data on the development data in order to make the data distribution shift smaller.

### Knowledge-driven methodologies that are transportable to specific target population

Seven studies focused on knowledge-driven methodology, all of which are transportable to specific target population. The methodology leverages external knowledge about causal relationships to understand whether predictors are fixed or vary across populations. For example, established risk factors of CVD such as age, smoking or statins are consistent in terms of the causal effect across different settings, but how these predictors are observed can vary. Each GPs could have different patterns of statins prescription, or different standardisation of patient’s smoking history. Understanding how predictors vary across populations such as statin prescription or smoking history previously discussed could make a CPM more transportable to specific population. For CVD, focusing on factors such as age, statins or smoking, and adjusting for local variations in how those predictors are observed by methods such as standardisation could potentially improve transportability to specific target population [47]. Crucially, this collection of methods assumes that researchers have prior knowledge regarding which predictors are stable and which vary across settings before the model is developed. Here, stability refers specifically to the invariance of the conditional distribution of the outcome given the relevant predictors across populations or environments, rather than to the prevalence or marginal distribution of the predictors being the same across populations.

#### Transportability formula and S-admissibility

These studies built on the structural causal model’s framework, which represents causal relationships among predictors and outcomes in forms of a causal diagram – a directed acyclic graph (DAG), and a set of mathematical equations describing such relationships. Pearl and Bareinboim [45] formalised transportability formulas for causal effects, noting that an identification of a given statistical relation, such as the conditional probability of Y given X, in the target population could be derived from a slightly modified version of the formula.

For example, when estimating CVD risk in a development population for application in a target population, it is assumed that both populations share a common set of predictors with stable causal relationships, including smoking and statin use. This implies that the risk of CVD, conditioned on smoking and statin use, is independent of the population selection—a property formally known as S-admissibility. In other words, the conditional risk of CVD given smoking and statins in the development population is identical to that in the target population. Developing a CVD prediction model satisfying S-admissibility is expected to be generalisable. A formal description of S-admissibility is provided in the Appendix.

#### Selection diagram

**Fig. 3:**
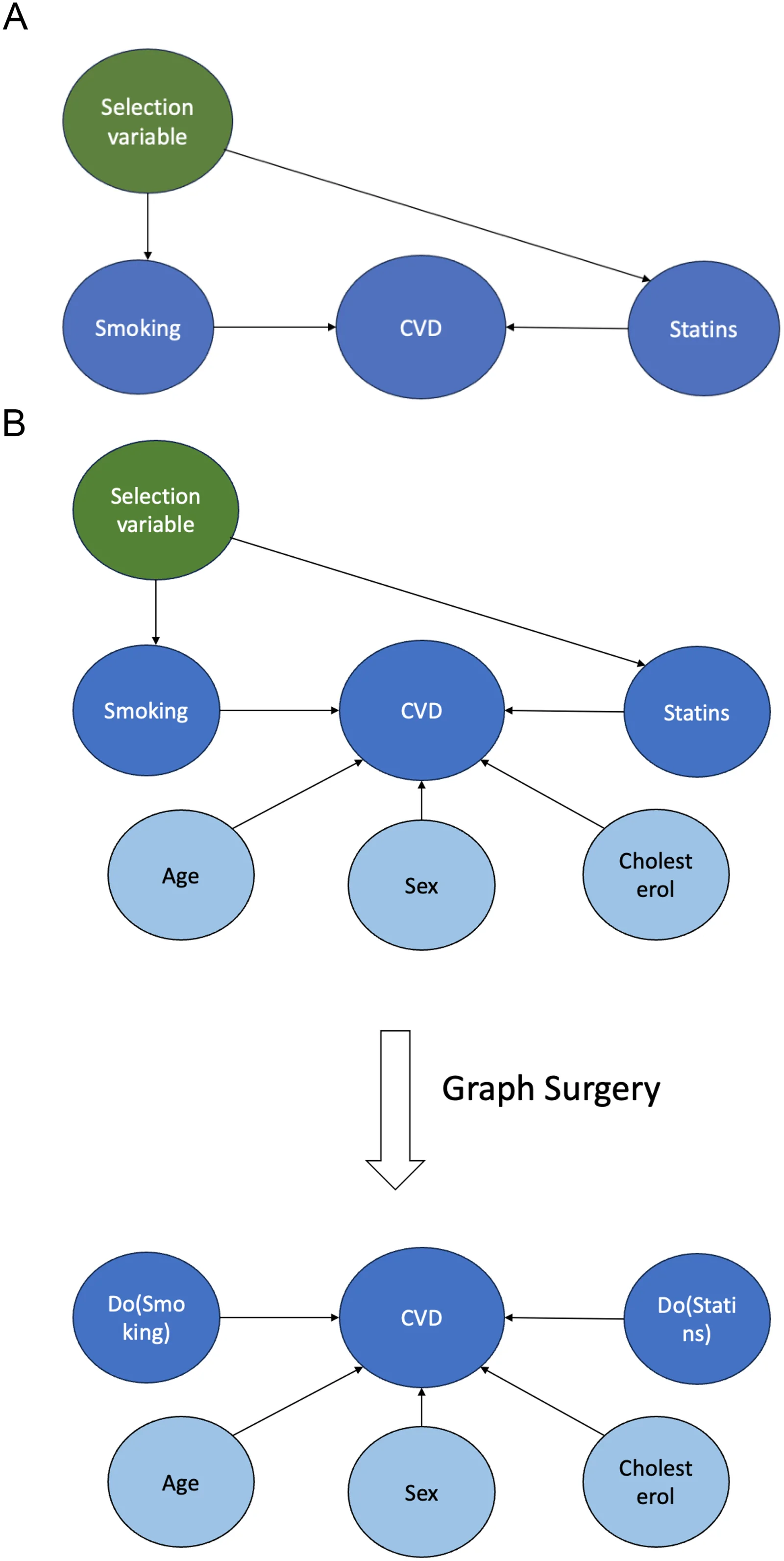
a: A selection diagram showing factors causing CVD. b: A selection diagram showing interventional distribution of factors causing CVD using “Graph Surgery”; do(smoking) and do(statins) represent hypothetical assignments of individual smoking and statins statuses, thus removing the influence of the selection variable

The concept of S-admissibility can be further extended to create a selection diagram (Fig. 3). It is a DAG having at least one selection variable, which is a variable that determines whether an individual belongs to the development or target population. A selection variable can affect or can be affected by other variables. S-admissibility of a set of predictors is satisfied if the outcome of interest is independent of the selection variables, conditional on the predictors. For example, suppose that we have developed a CVD risk prediction model using smoking and statins as predictors. However, in the target population there are less smokers and doctors are less likely to give statins(Fig. 3a). The set of predictors containing smoking and statins are S-admissible if the risk of CVD are equal between the development and target populations given the both covariate. That is, there are no other causes of CVD that also differ in both populations. In this setting, prediction of CVD using smoking and statins as predictors can be transported to the target population.

#### Graph surgery estimator

Subbaswamy and colleagues extended selection diagram for the Graph Surgery estimator [21]. The estimator employs causal algorithms to identify any combination of predictors that renders the do-operations on selection variables identifiable from observational data. This process effectively removes population-specific influences, resulting in a model that is more generalisable across diverse populations. The predictor set that yields the highest predictive performance is selected to form the final Graph Surgery estimator.

For example, a generalisable CVD risk prediction model where smoking and statin use are selection variables can be developed using the graph surgery estimator. In this scenario, the estimator identifies a specific subset of predictors—such as age, BMI, sex, and cholesterol levels—that satisfies the formal conditions for causal identification (Fig. 3b). By conditioning on this subset, the do-operations do(smoking) and do(statin)—which represent hypothetical scenarios where these variables are set to specific values—become identifiable from observational data. This process effectively performs “graph surgery” by severing the causal arrows originating from the selection variables that point toward smoking and statins. Consequently, the resulting model is free from population selection-induced influence, ensuring that predictions are driven by stable causal relationships rather than site-specific influence. This renders the model truly generalisable to new, unseen populations.

Subbaswamy et al. evaluated the Graph Surgery Estimator against other potential methods through two sets of experiments. In simulated linear Gaussian experiments, the surgery estimator demonstrated superior stability, maintaining a near-constant MSE in various scenarios where both Ordinary Least Squares (OLS) and graph pruning failed. When applied to real-world UCI Bike Sharing data, the surgery estimator achieved the lowest MSE in several test cases and consistently outperforming OLS during high-volatility seasonal shifts. Overall, their findings suggest that graph surgery is more stable than OLS and graph pruning across various dataset shift scenarios (Supplemental Table 1, No. 14).

#### Shortcut predictor

In addition to the selection diagram, Bellamy et al. proposed the characterisation of a shortcut predictor (feature) on a DAG [26]. A shortcut predictor is any predictor that is associated with an outcome and is site-specific. For example, a watermark in an electrocardiogram (ECG) used for diagnosing CVD is specific to each hospital. Identifying and removing shortcut predictors require expert knowledge on causal relationships of them and true predictors. Excluding the shortcut predictor before training a CPM is necessary for it to be generalisable. The shortcut predictor is essentially the same idea as the selection diagram previously described.

#### Minimising worse-case loss with mutable and immutable sets

This strategy leverages external knowledge to identify a set of predictors that adhere to the invariant property of the prediction system. This property is characterised by elements within the system that remain consistent, or invariant, across various populations, represented by immutable variables, and other elements that change across the system, represented by mutable variables. For example, when developing a CVD risk predictor using multi-centre data, we expect that the patient’s risk factors such as age, gender or family history are similar – or immutable (if we assume that each centre represents the same distribution), however, the patterns of treatments such as statins prescription might be different – or mutable – due to each centre’s policies. In this case the selection of variable should be directed toward immutable rather than mutable ones. In a paper by Subbaswamy et al., the authors proposed a method to identify the model that minimises the expected loss in the worst-case subpopulation, which is delineated by the sets of mutable and immutable variables [39]. The authors acknowledged that this method has the potential to guide the selection of generalisable prediction models that are stable to data distribution shift. However, they did not clearly explain how to achieve this. Further research could address this point. In addition, as a selection variable defines a variables that has structural difference in populations, which is a mutable variables, potential research could be done to explore how tackling generalisability and/or transportability by surgery estimator would achieve similar model performance as minimising worse-case loss with mutable and immutable sets does.

In addition, Liu (2023) adopted causal reasoning to distinguish between mutable and immutable variables [38]. Liu utilised minimax optimisation techniques based on these variable types and the specified causal graph to find an optimal subset of covariates that ensures stability, measured by the worst-case risk when transferring between populations. This subset serves as the starting point for developing generalisable and transportable models, however, similar to the previous methods from Subbaswamy et al., further research are needed on optimal methodology to develop a prediction model on this subset.

#### Causal predictor selection

**Fig. 4:**
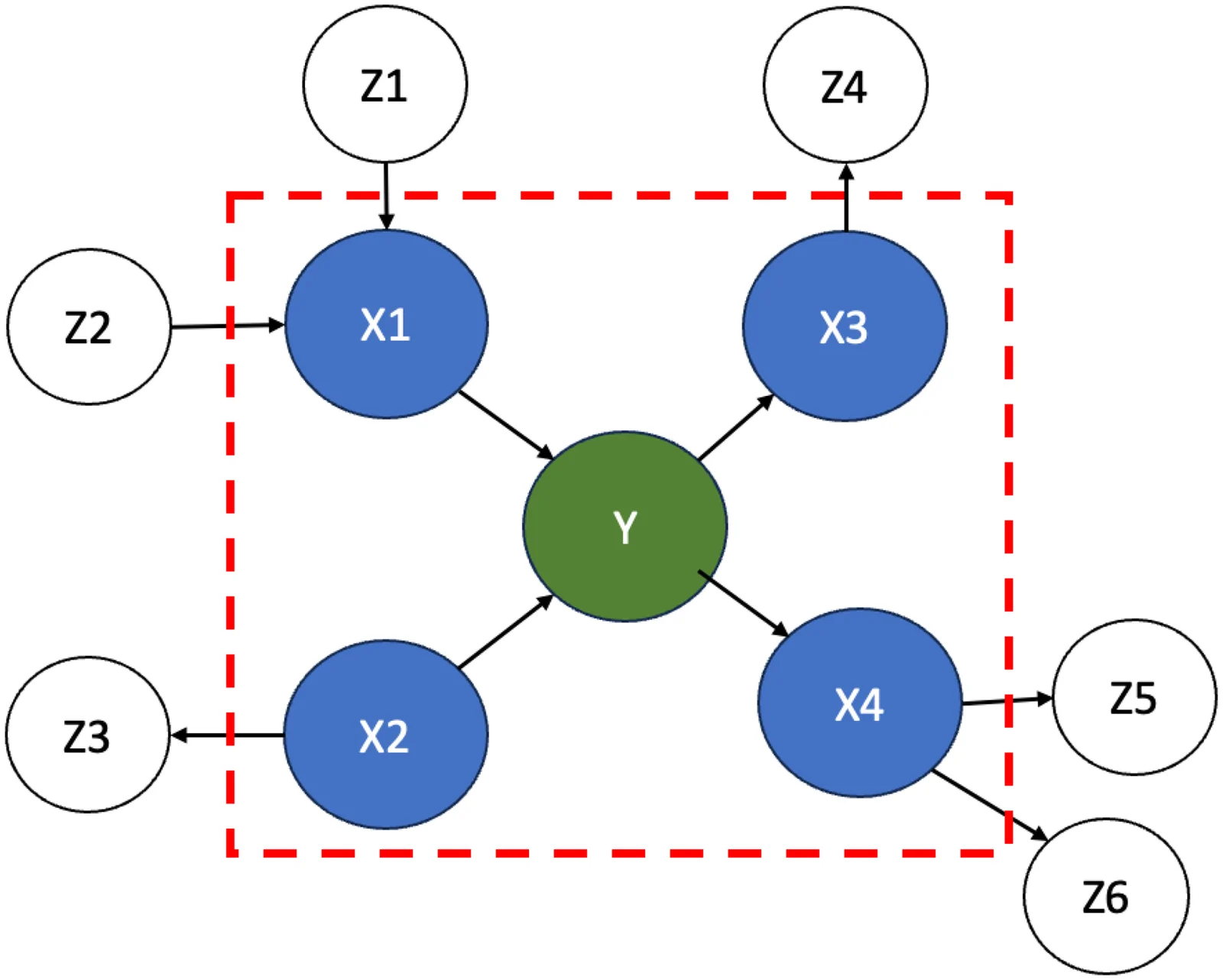
Prediction of Y with only variables that reside in Markov Blanket (red dash square)

Lemmon et al. applied predictor selection strategy motivated by causal structure(46). This is done by identifying predictors that reside in the Markov Blanket (MB) and those that are parents or children (Parent Child - PB) of the outcome. Based on a DAG incorporating causal relationships among predictors and an outcome, the Markov Blanket (MB) is a subset of the DAG that shields the outcome variable from other predictors (Fig. 4). That is, when we know all values of predictors residing in the MB, we will have all the information to predict the outcome, regardless of other predictors. Lemmon termed selection criterion with MB or PB as causal predictor selection, which utilises causal knowledge to correctly choose variables which are MB or PB, thereby orienting it more towards a knowledge-driven approach. Similarly to the predictor selection methods, selecting predictors causally related to the outcome, either through MB or PB, contributes to improved generalisability by making the model more parsimonious. This approach also reduces the chance of selecting variables that vary across different settings. This strategy was also done by Piccininni (2020), who formalised the approach that, based on a prescribed causal graph, the subset of predictors needs to be chosen only from those that are parents (causes) of the outcome, not the children (consequences) [27]. This is because the probability of a consequence given its cause is invariant across any populations, while the probability of a cause given its consequence is not. The scenario that is applicable in this context is that we are predicting disease outcomes based on their risk factors – prognostic prediction. However, this methodology is not useful in diagnostic prediction where the parents, i.e. disease of interest, are predicted based on the children, i.e. symptoms.

#### Causality-aware prediction by counterfactual approach

**Fig. 5:**
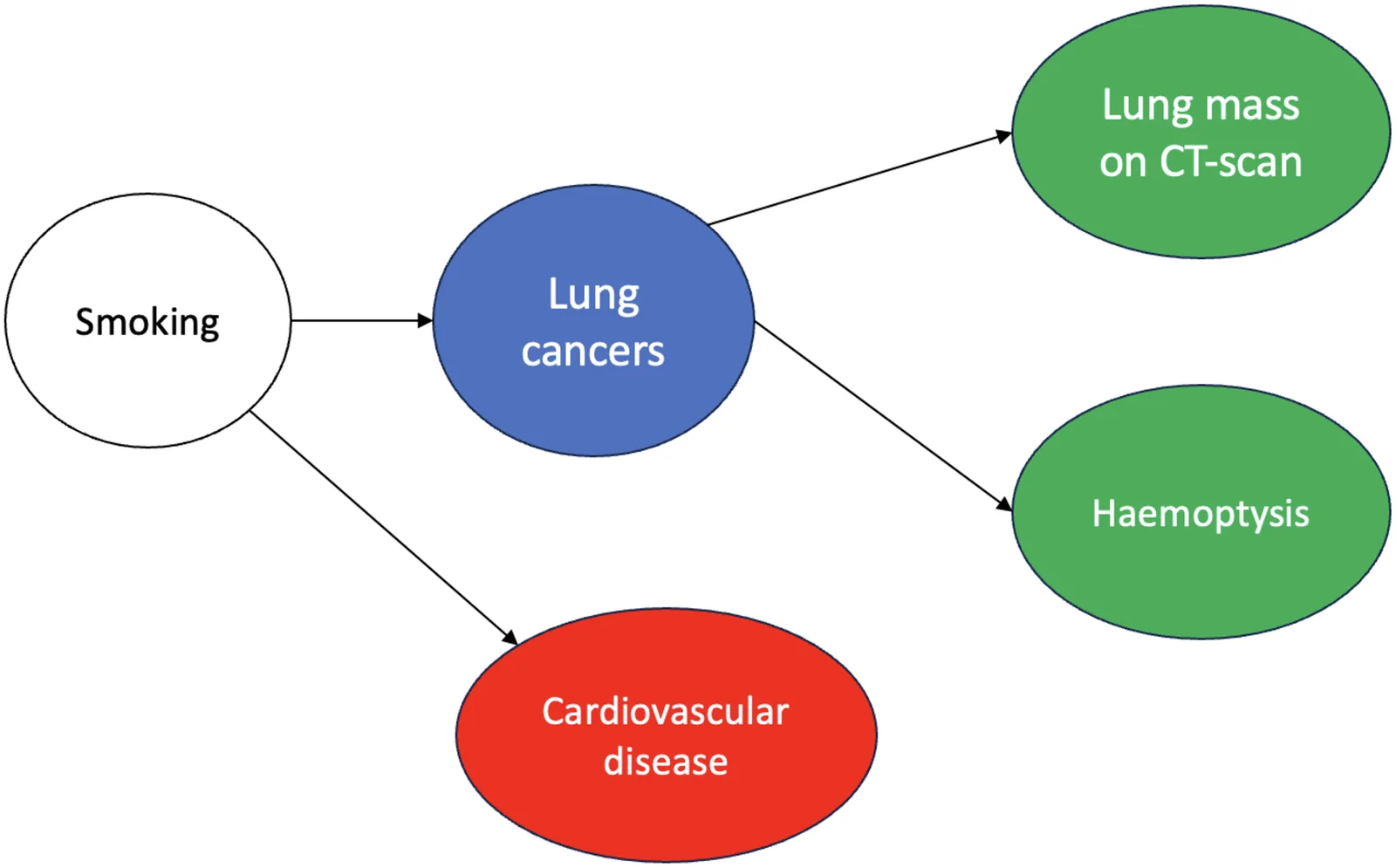
Diagnostic prediction of lung cancers with lung mass on CT-scan and haemoptysis (green circles) with need to adjust for confounding variable, CVD (red), in the target population when there are different in the distribution of confounding variable among development and target populations

Neto et al. proposed a causality-aware prediction approach for anticausal prediction, which involves using the consequences (effects) of an outcome as predictors to predict the outcome (the cause) itself [44]. For example, it involves prediction of lung cancers having cough or chest radiography as predictors. This method aims to predict the outcome of interest by training the prediction model on the simulated counterfactual predictors including only those that are direct effects of the outcome. For example, the authors simulated counterfactual data containing only the associations generated by the direct effect of the outcome (which is the effect of interest). It is particularly important when confounding is unstable between development and target populations. Interestingly, they found that the models that were adjusted for confounding using only development data are not robust for prediction in the target data when dataset shifts generated by selection bias is present. To improve the model’s robustness and thus the transportability to the target population, they suggested that the data from the target population is required to adjust for confounding on the target population. For example, in diagnostic prediction of lung cancers having lung mass on CT scan and haemoptysis as predictors, one need to adjust CVD in the target population, when the disease distribution is different in the target population. This is because confounding occurs as CVD and lung cancers share common cause (smoking), (Fig. 5). However, the authors examined the method on diagnostic prediction (e.g. prediction of CVD with chest pain and ECG test as predictors), which involve an anticausal direction. Causality-aware prediction for prognostic model on causal direction where predictors are causes (e.g. prediction of CVD with smoking and statins as predictors) may not have the same behaviour as Piccininni suggested [27].

## Discussion

### Summary of main findings

We have categorised approaches to aid the generalisability and transportability of clinical prediction models into two themes: one theme is data-driven, aimed at enhancing model robustness and only informed by empirical data. The other theme is knowledge-driven, which incorporates external knowledge in a form of causal relationships before using development data to train a clinical prediction model. The approaches can also be categorised according to whether they additionally take data from a target population during model development. Taking data from the target population as part of model development aids transportability to that specific target population. Models that do not use data from any target population focus on generalisability to a range of possible target populations.

For the data-driven approach, the concept of developing generalisable prediction models by increasing heterogeneity during development (enhancing between-cluster heterogeneity) and validation (utilising internal-external validation) was also previously proposed by Debray et al. [4], however, their paper was not included in this review as it did not match our search keywords. Bareinboim and Pearl had previously established a formal definition for the transportability of experimental findings from heterogeneous development population regarding causal effects [48]. Connecting this work with their earlier work on observational transportability (definition 5 in [45]), which states that a statistical relation can be transported to a new domain by reconstructing it using data from the new domain, including only a subset of variables, there is an implication that this definition of observational transportability could be modified to formalise the transportability of predictions from a heterogeneous development population. This interpretation supports the proposals by both Debray and colleagues [4] and Van Calster et al. [49], and aligns with the findings of this scoping review that using heterogeneous development population is likely to aid generalisability [28, 35, 41]. Future research could significantly contribute to this area of development. For example, to improve the transportability of clinical prediction models constructed using stepwise internal-external validation (SEICV) [28] on heterogenous development population, the method can be integrated with µ-Transportability [48]. µ-Transportability defines the transportability of causal relations from heterogenous source domains. It establishes a mathematical and graphical framework for “meta-transportability,” which allows integration of findings from multiple, heterogeneous source populations to accurately estimate causal effects in a target population where only observational data is available. However, accessing heterogeneous data is not straightforward in real-world settings because of a variety of factors, including administrative, governance, privacy, or technical barriers. Large healthcare databases are a valuable repository of heterogenous data, yet they are not totally exempt from those concerns [50]. As an alternative, the ensemble methodology promotes diversity at model levels and can mitigate privacy and governance concerns since base learners can be trained locally, reducing the need to centralise patient data [35]. However, large healthcare databases could be employed with data anonymisation techniques or General Data Protection Regulation (GDPR) standards to improve patient’s privacy so that these ensembles’ advantages may be no longer be required. A model developed using highly heterogeneous data may fail to perform in a new population if the development data were too diverse [51]. This highlights the trade-off between capturing optimal heterogeneity and ensuring transportability to a new target population.

In addition to increasing heterogeneity, we also found some papers suggesting that the generalisability of a clinical prediction model could be improved by ensuring the model contains only important predictors [46, 52]. We did not include these papers in the current review as there were insufficient theoretical information to support how this method would improve generalisability and/or transportability. The hypothesis is that such model is parsimonious and would include only predictors that are invariant to the data distribution shift, thereby improving the generalisability and/or transportability [46]. These methods include (1) Regularisation [46], (2) Remove and Retrain (ROAR) [46] and (3) Shapley value [52]. Regularisation shrinks the predictor coefficients toward zero. It reduces model variance and hence overfitting to development data. However, there is no guarantee that taking shrunken coefficients fitted from development population would improve generalisability and transportability in target population. ROAR and Shapley value work similarly in that they identify important predictors based on a permutation-based importance score (ROAR), or how each predictor performs on prediction task against the average prediction task (Shapley). According to the original papers, regularisation and important predictor selection yielded generalisable prediction in some circumstances, however, it is still not clear how the methods improve generalisability or transportability.

Some of the methods rely on the probability of being sampled from the target population and development population [34, 40, 43]. However, when using the methods for transportability in non-overlapped target and development populations, the methods probably do not provide any additional benefit. This is because in non-overlapping populations, there is a structural violation of positivity between the development and the target populations and therefore it is not possible to correctly identify the probability, which could lead to misspecified models.

Turning to the knowledge-driven approach, it might initially seem to be a natural method for training generalisable or transportable prediction models, as it aims to capture and learn from causal structures that should theoretically aid generalisability and transportability. However, the process is not as straightforward as it appears. As the approach’s name implies, it entails leveraging domain knowledge of the underlying causal structure to construct structural causal models or graphs. This method is somewhat equivalent to estimating causal effect using randomised or observational studies, in such a way that it leverages domain knowledge to construct the underlying causal structure of the problems being studied. Though both requires assuming that the causal models are true, the causal effect estimation by randomisation have fewer causal assumptions, particularly on the study population. A common thread among the knowledge-driven methodologies reviewed in this study is the reliance on strong assumptions regarding accurately specified causal structures. This is particularly true for the specifications of selection variables or assumptions about specific covariates causing variations in the distributions between development and target populations. To best of our knowledge, no studies have yet proposed a methodology that estimates causal effects that is resistant to effect modifiers and use these effects to construct generalisable prediction models with minimal extra assumptions. There is a growing literature proposing methods to transport a causal effect to the target population in the presence of effect modifiers [33, 53, 54]. Lin et al. [55] identified causal methods that allow predictions under hypothetical interventions. We could view these interventions as situations in which an individual who is a member of development population would have been reassigned to the target population. This could possibly answer generalisability and transportability problems. Identifying causal methods to enable prediction under hypothetical interventions of “switching” the populations could be the next direction of methodological research on generalisability and transportability.

Addressing generalisability and transportability of CPMs from a data-driven perspective is not straightforward. Traditionally, developed models are externally validated in different populations and settings to ensure that they would perform as they did in development settings. Van Calster et al. argue that an external validation only captures generalisability and transportability on a single external dataset so says nothing about ‘generalisability in general’ [49]. However, the distribution of the population underlying the evaluating external dataset can change from the point at which the external validation is taken, to the point at which the model is being implemented, there is no reason to assume that models that have been evaluated with the external validation are always transportable. This is because though the models are perfectly validated in the static external validation, there is no guarantee that the models would be well performed in the new, changing environments [49]. Instead, they offer a series of recommendations for model development and validation that align with the data-driven methodology presented in this scoping review, which includes incorporating greater heterogeneity in the training and validation processes.

### Comparing with other studies

Recent studies have conducted methodological reviews on generalisability and transportability from various perspectives: one addresses the external validity of causal effects, including heterogeneity of treatment effect in terms of generalisability and transportability [56], while another focuses on generalising or transporting treatment effects from randomised clinical trials to target populations [31]. Furthermore, there exist the comprehensive surveys on transfer learning [57, 58], a subfield in machine learning that aims to enhance the learning of prediction models in the target population by utilising knowledge derived from models previously trained in the development population. This facilitates the transfer of knowledge from development to target. Although transfer learning and transportability are not equivalent, their methodologies share many similar characteristics that reveal some overlap between these domains. Accordingly, there are studies that have adopted transfer learning approaches, such as density ratio weighting, to improve the transportability of clinical prediction models or causal effects to a target population [34, 36, 40, 59–63]. While this current review did not include these methodologies, which were uncaptured with the search terms, it contributes to the body of knowledge on generalisability and transportability within predictive modelling by demonstrating that the methodologies aiding generalisability and transportability of clinical prediction models have been proposed from various fields of study. Previously, there are reviews on model updating, which is also essentially a form of improving the transportability of a CPM [13, 14].

### Strengths and limitations

To our knowledge, this review is the first to synthesise the key methodologies for aiding generalisability and transportability, specifically within the clinical prediction landscape. It also describes how these methodologies can be used for aiding generalisability or transportability of a CPM to target population. However, it has some limitations. As the studies it encompasses originated from two distinct communities: medical statistics and machine learning. Consequently, the terminologies employed to articulate the act of generalising or transporting findings from a development to a target population differ. This diversity presents a challenge in designing search terms that are comprehensive enough to capture relevant articles while excluding non-pertinent ones and avoiding the omission of seminal works. Martin et al. [25] addressed this issue by proposing an iterative search-and-refine strategy to optimally identify pertinent literature. Although this review has adhered to such an approach, the notable variation in language among both disciplines [64], raises the possibility that some relevant papers may have been missed. This became evident when one of the five known papers was not captured by the search terms and remained undetected through citation searching. Moreover, most exclusions took place during the abstract screening phase, notably omitting studies concerning the external validation of clinical prediction models. Abstracts from such studies frequently have insufficient detail to determine whether the methodologies employed were specifically designed to address transportability issues. Consequently, this could result in the inadvertent exclusion of relevant methodologies that only apparent upon full text review. This poses a significant challenge when screening a large volume of papers based solely on abstracts. Also, the data extraction was done only by the primary author, there will be no independent verification of the extracted data, which may affect the reliability of the findings. Another limitation of the search strategy is that certain databases, such as Scopus or Web-of-Science were not included, which may have led to the missing of relevant studies, particularly those outside the medical field.

### Future research recommendations

This scoping review identifies several key gaps for future research to develop optimal methodology aiding generalisability and transportability of clinical prediction models. Firstly, methodologies that integrate data-driven techniques—like the use of heterogeneous data—with robust causal approaches could be particularly useful. For example, the application of counterfactual predictions from observational data may be a step in this direction. Secondly, as several methodologies necessitate access to target population data, which have the strengths of transporting a prediction model to specific target population, there is a need for methods that reduce this requirement but still retains the ability for the transportability to the population of interest. This may include creating hypothetical population where the distribution is assumed to be similar as the target population. Thirdly, since our review focused on structured data, further research exploring unstructured data—such as clinical prediction using images, text, or generative AI—would be useful. Finally, and the most importantly, there is a need to evaluate the performance of these proposed methodologies on both simulated and real-world datasets that present various types of dataset shifts, to understand their efficacy in broader contexts.

## Conclusions

In conclusion, this scoping review suggests that methodology to aid generalisability and transportability of clinical prediction models can be categorised as data-driven and knowledge-driven. Data-driven methods focus on harnessing actual data to maximise model robustness, including heterogeneity or importance weighting. In contrast, knowledge-based methods utilise causal reasoning to form underlying invariant structure that encoding generalisable or transportable prediction models. Future work could explore integrating both approaches into a single, unified one for better generalisability and transportability.

## Supplementary Information

Below is the link to the electronic supplementary material.

Supplementary Material 1

Supplementary Material 2

Supplementary Material 3

## Data Availability

No datasets were generated or analysed during the current study.

## References

[1] Steyerberg EW. Clinical prediction models: a practical approach to development, validation, and updating. 2nd ed. Cham, Switzerland: Springer; 2019. (Statistics for Biology and Health). 10.1007/978-3-030-16399-0.

[2] Riley RD, van der Windt D, Croft P, Moons KGM, editors. Prognosis research in healthcare: concepts, methods, and impact [Internet]. Oxford University Press. 2019 [cited 2023 May 2]. Available from: 10.1093/med/9780198796619.001.0001.

[3] Justice AC, Covinsky KE, Berlin JA. Assessing the Generalizability of Prognostic Information. Ann Intern Med. 1999;130(6):515–24. 10.7326/0003-4819-130-6-199903160-00016. 10075620

[4] Debray TPA, Moons KGM, Ahmed I, Koffijberg H, Riley RD. A framework for developing, implementing, and evaluating clinical prediction models in an individual participant data meta-analysis. Stat Med. 2013;32(18):3158–80. 10.1002/sim.5732. 23307585

[5] Collins GS, Dhiman P, Ma J, Schlussel MM, Archer L, Calster BV, et al. Evaluation of clinical prediction models (part 1): from development to external validation. BMJ. 2024;384:e074819. 10.1136/bmj-2023-074819. PubMed PMID: 38191193. PMC10772854 38191193

[6] Riley RD, Archer L, Snell KIE, Ensor J, Dhiman P, Martin GP, et al. Evaluation of clinical prediction models (part 2): how to undertake an external validation study. BMJ. 2024;384:e074820. 10.1136/bmj-2023-074820. PubMed PMID: 38224968. PMC10788734 38224968

[7] Sperrin M, Riley RD, Collins GS, Martin GP. Targeted validation: validating clinical prediction models in their intended population and setting. Diagn Progn Res. 2022;6(1):24. 10.1186/s41512-022-00136-8. PMC9773429 36550534

[8] Wynants L, Calster BV, Collins GS, Riley RD, Heinze G, Schuit E, et al. Prediction models for diagnosis and prognosis of covid-19: systematic review and critical appraisal. BMJ. 2020;369:m1328. 10.1136/bmj.m1328. PubMed PMID: 32265220. PMC7222643 32265220

[9] Quiñonero-Candela J, Sugiyama M, Schwaighofer A, Lawrence ND, editors. Dataset shift in machine learning [Internet]. The MIT Press; 2008 [cited 2023 Aug 29]. Available from: 10.7551/mitpress/9780262170055.001.0001.

[10] Subbaswamy A, Saria S. From development to deployment: dataset shift, causality, and shift-stable models in health AI. Biostatistics. 2020;21(2):345–52. 10.1093/biostatistics/kxz041. 31742354

[11] Finlayson SG, Subbaswamy A, Singh K, Bowers J, Kupke A, Zittrain J, et al. The Clinician and Dataset Shift in Artificial Intelligence. N Engl J Med. 2021;385(3):283–6. 10.1056/NEJMc2104626. PMC8665481 34260843

[12] Lenert MC, Matheny ME, Walsh CG. Prognostic models will be victims of their own success, unless. J Am Med Inform Assoc. 2019 Dec 1;26(12):1645–50. 10.1093/jamia/ocz145. PMC6857506 31504588

[13] Su TL, Jaki T, Hickey GL, Buchan I, Sperrin M. A review of statistical updating methods for clinical prediction models. Stat Methods Med Res. 2018;27(1):185–97. 10.1177/0962280215626466. 27460537

[14] Jenkins DA, Sperrin M, Martin GP, Peek N. Dynamic models to predict health outcomes: current status and methodological challenges. Diagn Progn Res. 2018;2(1):23. 10.1186/s41512-018-0045-2. PMC6460710 31093570

[15] Steyerberg EW, Vickers AJ, Cook NR, Gerds T, Gonen M, Obuchowski N, et al. Assessing the Performance of Prediction Models: A Framework for Traditional and Novel Measures. Epidemiology. 2010;21(1):128. 10.1097/EDE.0b013e3181c30fb2. PMC3575184 20010215

[16] Janssen KJM, Moons KGM, Kalkman CJ, Grobbee DE, Vergouwe Y. Updating methods improved the performance of a clinical prediction model in new patients. J Clin Epidemiol. 2008;61(1):76–86. 10.1016/j.jclinepi.2007.04.018. 18083464

[17] Debray TPA, Koffijberg H, Vergouwe Y, Moons KGM, Steyerberg EW. Aggregating published prediction models with individual participant data: a comparison of different approaches. Stat Med. 2012;31(23):2697–712. 10.1002/sim.5412. 22733546

[18] Debray TPA, Koffijberg H, Nieboer D, Vergouwe Y, Steyerberg EW, Moons KGM. Meta-analysis and aggregation of multiple published prediction models. Stat Med. 2014;33(14):2341–62. 10.1002/sim.6080. 24752993

[19] Hickey GL, Grant SW, Caiado C, Kendall S, Dunning J, Poullis M, et al. Dynamic Prediction Modeling Approaches for Cardiac Surgery. Circ Cardiovasc Qual Outcomes. 2013;6(6):649–58. 10.1161/CIRCOUTCOMES.111.000012. 24150044

[20] McCormick TH, Raftery AE, Madigan D, Burd RS. Dynamic Logistic Regression and Dynamic Model Averaging for Binary Classification. Biometrics. 2012;68(1):23–30. 10.1111/j.1541-0420.2011.01645.x. PMC3831847 21838812

[21] Subbaswamy A, Schulam P, Saria S. Preventing failures due to dataset shift: learning predictive models that transport. In: Proceedings of the Twenty-Second International Conference on Artificial Intelligence and Statistics [Internet]. PMLR; 2019 [cited 2023 Jun 23]. p. 3118-3127. Available from: https://proceedings.mlr.press/v89/subbaswamy19a.html

[22] Fehr J, Piccininni M, Kurth T, Konigorski S. A causal framework for assessing the transportability of clinical prediction models [Internet]. medRxiv; 2022 [cited 2023 Feb 1];2022.03.01.22271617. Available from: https://www.medrxiv.org/content/. 10.1186/s12874-023-02003-6 PMC10439645 37598141

[23] Kaddour J, Lynch A, Liu Q, Kusner MJ, Silva R. Causal machine learning: a survey and open problems [Internet]. 2022. Available from: https://arxiv.org/abs/2206.15475.

[24] Page MJ, McKenzie JE, Bossuyt PM, Boutron I, Hoffmann TC, Mulrow CD, et al. The PRISMA 2020 statement: an updated guideline for reporting systematic reviews. BMJ. 2021;372:n71. 10.1136/bmj.n71. PubMed PMID: 33782057. PMC8005924 33782057

[25] Martin GP, Jenkins DA, Bull L, Sisk R, Lin L, Hulme W, et al. Toward a framework for the design, implementation, and reporting of methodology scoping reviews. J Clin Epidemiol. 2020;127:191–7. 10.1016/j.jclinepi.2020.07.014. 32726605

[26] Bellamy D, Hernán MA, Beam A. A structural characterization of shortcut features for prediction. Eur J Epidemiol. 2022;37(6):563–8. 10.1007/s10654-022-00892-3. PMC9256901 35792990

[27] Piccininni M, Konigorski S, Rohmann JL, Kurth T. Directed acyclic graphs and causal thinking in clinical risk prediction modeling. BMC Med Res Methodol. 2020;20(1):179. 10.1186/s12874-020-01058-z. PMC7331263 32615926

[28] de Jong VMT, Moons KGM, Eijkemans MJC, Riley RD, Debray TPA. Developing more generalizable prediction models from pooled studies and large clustered data sets. Stat Med. 2021;40(15):3533–59. 10.1002/sim.8981. PMC8252590 33948970

[29] Geersing GJ, Bouwmeester W, Zuithoff P, Spijker R, Leeflang M, Moons K. Search Filters for Finding Prognostic and Diagnostic Prediction Studies in Medline to Enhance Systematic Reviews. PLoS ONE. 2012;7(2):e32844. 10.1371/journal.pone.0032844. PMC3290602 22393453

[30] Colnet B, Mayer I, Chen G, Dieng A, Li R, Varoquaux G, et al. Causal inference methods for combining randomized trials and observational studies: a review [Internet]. 2020 Nov 16. 10.48550/arXiv.2011.08047. PMC12499922 41059256

[31] Ling AY, Montez-Rath ME, Carita P, Chandross KJ, Lucats L, Meng Z, et al. An Overview of current methods for real-world applications to generalize or transport clinical trial findings to target populations of interest. Epidemiology. 10.1097/EDE.0000000000001633. PMC10392887 37255252

[32] Kallus N, Puli AM, Shalit U. Removing hidden confounding by experimental grounding. in: advances in neural information processing systems [Internet]. Curran Associates, Inc.; 2018 [cited 2023 May 11]. Available from: https://papers.nips.cc/paper_files/paper/2018/hash/566f0ea4f6c2e947f36795c8f58ba901-Abstract.html.

[33] Dahabreh IJ, Robertson SE, Steingrimsson JA, Stuart EA, Hernán MA. Extending inferences from a randomized trial to a new target population. Stat Med. 2020;39(14):1999–2014. 10.1002/sim.8426. 32253789

[34] Gao J, Mar PL, Chen G. More generalizable models for sepsis detection under covariate shift. amia jt summits. Transl Sci Proc AMIA Jt Summits Transl Sci. 2021;2021:220–8. PubMed PMID: 34457136; PubMed Central PMCID: PMC8378628. PMC8378628 34457136

[35] Reps JM, Williams RD, Schuemie MJ, Ryan PB, Rijnbeek PR. Learning patient-level prediction models across multiple healthcare databases: evaluation of ensembles for increasing model transportability. BMC Med Inform Decis Mak. 2022;22(1):142. Located at: Embase; 638097644. 10.1186/s12911-022-01879-6. PMC9134686 35614485

[36] Dockès J, Varoquaux G, Poline JB. Preventing dataset shift from breaking machine-learning biomarkers. GigaScience. 2021;10(9):giab055. 10.1093/gigascience/giab055. PMC8478611 34585237

[37] Pajouheshnia R, van Smeden M, Peelen LM, Groenwold RHH. How variation in predictor measurement affects the discriminative ability and transportability of a prediction model. J Clin Epidemiol. 2019;105:136–41. 10.1016/j.jclinepi.2018.09.001. 30223065

[38] Liu M, Zheng X, Sun X, Fang F, Wang Y. Which invariance should we transfer? a causal minimax learning approach [Internet]. arXiv; 2023 [cited 2023 Sep 25]. Available from: http://arxiv.org/abs/2107.01876.

[39] Subbaswamy A, Adams R, Saria S. Evaluating model robustness and stability to dataset shift [Internet]. arXiv; 2021 [cited 2023 Sep 25]. Available from: http://arxiv.org/abs/2010.15100.

[40] Steingrimsson JA, Gatsonis C, Li B, Dahabreh IJ. Transporting a Prediction Model for Use in a New Target Population. Am J Epidemiol. 2023;192(2):296–304. 10.1093/aje/kwac128. PMC11004796 35872598

[41] Schinkel M, Bennis FC, Boerman AW, Wiersinga WJ, Nanayakkara PWB. Embracing cohort heterogeneity in clinical machine learning development: a step toward generalizable models. Sci Rep. 2023;13(1):8363. 10.1038/s41598-023-35557-y. PMC10209202 37225751

[42] Gong JJ, Sundt TM, Rawn JD, Guttag JV. Instance weighting for patient-specific risk stratification models. In: Proceedings of the 21th ACM SIGKDD International Conference on Knowledge Discovery and Data Mining [Internet]. New York, NY, USA: Association for Computing Machinery; 2015 [cited 2023 Nov 15]. pp. 369–78. (KDD ’15). Available from: 10.1145/2783258.2783397.

[43] Qian H, Wang B, Ma P, Peng L, Gao S, Song Y. Managing dataset shift by adversarial validation for credit scoring [Internet]. arXiv; 2021 [cited 2023 Sep 25]. Available from: http://arxiv.org/abs/2112.10078.

[44] Neto EC. Towards causality-aware predictions in static anticausal machine learning tasks: the linear structural causal model case [Internet]. arXiv; 2020 [cited 2023 Sep 25]. Available from: http://arxiv.org/abs/2001.03998.

[45] Pearl J, Bareinboim E. Transportability of causal and statistical relations: a formal approach. In: 2011 IEEE 11th International Conference on Data Mining Workshops [Internet]. 2011 [cited 2023 Nov 15]. pp. 540–7. Available from: https://ieeexplore.ieee.org/document/6137426.

[46] Lemmon J, Guo LL, Posada J, Pfohl SR, Fries J, Fleming SL, et al. Evaluation of Feature Selection Methods for Preserving Machine Learning Performance in the Presence of Temporal Dataset Shift in Clinical Medicine. Methods Inf Med. 2023;62(1–2):60–70. 10.1055/s-0043-1762904. Located at: Embase; 640392008. 36812932

[47] Lasko TA, Strobl EV, Stead WW. Why do probabilistic clinical models fail to transport between sites. Npj Digit Med. 2024;7(1):1–8. 10.1038/s41746-024-01037-4. PMC10907678 38429353

[48] Bareinboim E, Pearl J. Meta-transportability of causal effects: a formal approach. In: Proceedings of the Sixteenth International Conference on Artificial Intelligence and Statistics [Internet]. PMLR; 2013 [cited 2023 Nov 15]. pp. 135–43. Available from: https://proceedings.mlr.press/v31/bareinboim13a.html.

[49] Van Calster B, Steyerberg EW, Wynants L, van Smeden M. There is no such thing as a validated prediction model. BMC Med. 2023;21(1):70. 10.1186/s12916-023-02779-w. PMC9951847 36829188

[50] Riley RD, Ensor J, Snell KIE, Debray TPA, Altman DG, Moons KGM, et al. External validation of clinical prediction models using big datasets from e-health records or IPD meta-analysis: opportunities and challenges. BMJ. 2016;353:i3140. 10.1136/bmj.i. 3140 PubMed PMID: 27334381. PMC4916924 27334381

[51] Peek N, Hindricks G, Akbarov A, Tijssen JGP, Jenkins DA, Kapacee Z, et al. Sudden cardiac death after myocardial infarction: individual participant data from pooled cohorts. Eur Heart J. 2024;45(43):4616–26. 10.1093/eurheartj/ehae326. PMC11560274 39378245

[52] Sebastián C, González-Guillén CE. A feature selection method based on Shapley values robust to concept shift in regression [Internet]. arXiv; 2023 [cited 2023 Sep 25]. Available from: http://arxiv.org/abs/2304.14774.

[53] Westreich D, Edwards JK, Lesko CR, Stuart E, Cole SR. Transportability of trial results using inverse odds of sampling weights. Am J Epidemiol. 2017;186(8):1010–4. Located at: Embase; 618914727. 10.1093/aje/kwx164. PMC5860052 28535275

[54] Rudolph KE, Laan MJ. Robust Estimation of Encouragement Design Intervention Effects Transported Across Sites. J R Stat Soc Ser B Stat Methodol. 2017;79(5):1509–25. 10.1111/rssb.12213. PMC5784860 29375249

[55] Lin L, Sperrin M, Jenkins DA, Martin GP, Peek N. A scoping review of causal methods enabling predictions under hypothetical interventions. Diagn Progn Res. 2021;5(1):3. 10.1186/s41512-021-00092-9. PMC7860039 33536082

[56] Degtiar I, Rose S. A Review of Generalizability and Transportability. Annu Rev Stat Its Appl. 2023;10(1):501–24. 10.1146/annurev-statistics-042522-103837.

[57] Pan SJ, Yang Q. A Survey on Transfer Learning. IEEE Trans Knowl Data Eng. 2010;22(10):1345–59. 10.1109/TKDE.2009.191.

[58] Weiss K, Khoshgoftaar TM, Wang D. A survey of transfer learning. J Big Data. 2016;3(1):9. 10.1186/s40537-016-0043-6.

[59] Colnet B, Josse J, Varoquaux G, Scornet E. Causal effect on a target population: A sensitivity analysis to handle missing covariates. J Causal Inference. 2022;10(1):372–414. 10.1515/jci-2021-0059.

[60] Nie X, Imbens G, Wager S. Covariate balancing sensitivity analysis for extrapolating randomized trials across locations [Internet]. arXiv; 2021 [cited 2023 Nov 15]. Available from: http://arxiv.org/abs/2112.04723.

[61] Han L, Hou J, Cho K, Duan R, Cai T. Federated adaptive causal estimation (FACE) of target treatment effects [Internet]. arXiv; 2023 [cited 2023 Nov 15]. Available from: http://arxiv.org/abs/2112.09313. 10.1080/01621459.2025.2453249 PMC12396575 40895281

[62] van der Kyono T. Exploiting Causal Structure for Robust Model Selection in Unsupervised Domain Adaptation. IEEE Trans Artif Intell. 2021;2(6):494–507. 10.1109/TAI.2021.3101185.

[63] Uehara M, Kato M, Yasui S. Off-policy evaluation and learning for external validity under a covariate shift. In: advances in neural information processing systems [internet]. curran associates, Inc.; 2020 [cited 2023 Nov 15]. pp. 49–61. Available from: https://proceedings.neurips.cc/paper/2020/hash/0084ae4bc24c0795d1e6a4f58444d39b-Abstract.html.

[64] Wan B, Caffo B, Vedula SS. A Unified Framework on Generalizability of Clinical Prediction Models. Front Artif Intell. 2022;5:872720. 10.3389/frai.2022.872720. PMC9100692 35573904

