# Supplementary material for: Scoping review of methodology for aiding generalisability and transportability of clinical prediction models
Source: Diagn Progn Res. 2026 Oct 1;10:25. doi: 10.1186/s41512-026-00237-8 (PMC13629139; doi:10.1186/s41512-026-00237-8)
Supplement: Supplementary file 1 [file 41512_2026_237_MOESM1_ESM.docx]

Supplemental table 1: Summary of included 18 papers

| **No** | **Authors** | **Methodology** | **Description** | **Transportable to specific population** | **Domain knowledge** | **Stated assumptions** | **Reported advantages** | **Reported limitations** | **Applied examples** |
| --- | --- | --- | --- | --- | --- | --- | --- | --- | --- |
| 1 | Gao et al. (34) | (D)  Density ratio weighing | These are the methods that were used to estimate the density ratio: 1. Direct methods - KMM and RuLSIF 2. Indirect methods - LR with L1 and L2 penalty, and Random Forests | Yes, note that outcome data is required only for the indirect methods | No | The distribution shift is compatible with covariate shift | Estimation of density ratio with both parametric and non-parametric models are useful for covariate shift correction. | Using density ratio weighting is not possible to correct concept shift. | CPMs with weighted likelihood estimation using these odds were fitted to predict sepsis in hospitalised patients. The study demonstrated that density ratio weighting significantly improved the generalisability of sepsis detection models under covariate shift. While discrimination (AUROC) consistently improved, calibration (Brier scores) did not show uniform improvement, particularly in complex models with extensive hyperparameters. |
| 2 | Liu et al. (38) | (D)  Predictor selection of invariant information from a causal perspective | The authors propose that selecting the entire set of stable predictors, S, is not always optimal, as retaining all stable information may not yield the best results. This is determined by a graphical condition based on the causal relationships encoded in the data, which can be verified through causal discovery methods. The condition specifically assesses whether the outcome variable significantly impacts a subset of mutable variables that are descendants of another set directly related to the target. If S is not deemed optimal, the authors introduce an optimisation algorithm to identify a subset of predictors with the minimal worst-case risk. This algorithm starts by learning a causal graph from the training data, encompassing the outcome and predictors. It then checks the influence of the outcome variable on the specified subset of variables to decide if the full set S is optimal. If not, the algorithm categorises predictors based on their risk profiles and selects the group with the lowest risk, ensuring the model's robustness across varying data conditions. | Yes | Yes | The methodology is based on a faithful  structural causal framework and assumes a Markovian structure. It assumes that the distributional shifts are only due to changes in the mutable variables' distribution. Though, correctly identifying them is particularly difficult when working with real-world data. | It introduces a novel optimisation  algorithm to estimate the worst-case risk for each subset of stable predictors and selects the one with the minimal risk. | The effectiveness of the methodology is dependent on the accuracy of the assumed causal framework, including the specification of mutable and immutable variables. In addition, it requires computational resources for causal discovery and the evaluation of worst-case risks. | The authors tested their causal minimax learning approach against various baselines such as Vanilla, ICP, IC, DRO, and others. It used metrics including max MSE and std MSE for evaluation. The synthetic study involved data generation across 20 different environments using a particular DAG and structural equation setup, where the distribution of a mutable variable varied. The authors also evaluated a predictive model for Alzheimer's disease, employing neuroimaging data to identify key predictors, with distinct environmental patterns observed across various age groups. |
| 3 | Subbaswamy et al.  (39) | (D) Robustness and stability assessment | - Classifying predictors: Classifying predictors into immutable and mutable sets. - Defining distribution shifts: Factoring the probability distribution to focus on the impact of changes in mutable variables' distributions. - Assessing Robustness: Evaluating worst-case risk by identifying subpopulations with the worst average loss under distribution shifts. | Yes | Yes, to define mutable/immutable variables  and formulate distribution shift | - The subpopulation identified for estimation of worst-case performance is representative of potential real-world shift - The mutable, immutable variables and their distributions are assumed correctly. | - Allow for estimate performance in worst-case populations, reflecting stability, without need to collect target data | Not stated | - Evaluated classical and robust models, for predicting sepsis with 17 predictors using EHR data from hospital A over four years - Compared estimated worst-case performance under difference in pattern of lab test ordering |
| 4 | Piccininni et al. (27) | (D, K)  Selecting predictors which is a parent (cause),  not a child (consequences) | The principle of independent mechanism states that P(cause), and P(effect\|cause) contain no information about each other, both are independent of each other but not the anti-direction. This independence makes effect prediction model with P(efffect\|cause) stable and transportable to another settings where P(effect) changes, i.e. covariate data shift. | No | Yes, to build a faithful DAG | DAG must be correctly specified (faithfulness) and contains no hidden variables. | The model is simple to understand. | DAG must be correctly specified (faithfulness) and contains no hidden variables. | There are no experiment involving how prediction selection with MB would impact transportability (the authors did simulated experiments, but mainly focused on internal validation of CPM with various predictor selection strategies). |
| 5 | Steingrimsson et al. (40) | (D)  Importance weighting by density ratio estimation | Correctly-specified Model: Tailoring the model for the target population is not required.   Misspecified Model: The tailoring involves estimating the density ratio, which is done by using the inverse of the odds of being from the development population as weights. These weights are then applied in the prediction model to adjust it for use in the target population. This step is crucial to account for differences in the distribution of covariates between the development and target populations. | Yes, and only covariate data from the target is required. | Yes | 1. Conditional independence of the outcome and the population: this requires that the outcome is independent of selection in the population conditional on covariates, which implies conditional mean exchangeability over the population. According to this assumption, the relationship between the outcome and covariates remains constant common across populations. 2. Positivity: this condition necessitates that for every covariate pattern present in the target population, there is a positive probability of encountering the same pattern in the development population. | The methodology aids tailoring a prediction model from the development population to the target population  by employing weighted adjustments to individual data points without the need for re-training the model. Additionally, these weighted approaches provide insights into the variations in the model's performance between the development and target populations. | 1. The applicability of the methods may be limited if there is a lack of access to covariate data from the target population. 2. The set of covariates necessary to satisfy the conditional independence assumption might be extensive than the set used in the model. 3. The approach did not address the practical issues such as missing data and measurement error. | The authors used simulated data to test the performance of the prediction models. Data was generated using a linear model with heteroscedastic errors and simulated membership for development population using logistic regression. They assessed both with Ordinary Least Squares (OLS) and Weighted Least Squares (WLS) regression. WLS incorporating weights based on inverse odds of being in the development data training sets. They also used real-world data for prediction of lung cancer diagnosis in the US using data from the NLST and the NHANES. A prediction model was tailored with demographic and health predictors using inverse-odds weighted logistic regression to transport a the trial (NLST) to a national setting (NHANES). |
| 6 | Schinkel et al. (41) | (D)  Induce training data heterogeneity | AI models trained on multiple cohorts with heterogeneity and diluted hospital-specific patterns, are more generalisable. | No | No | Heterogeneous cohort data is available. | - | - | Testing patients having blood cultures in ER in different medical centres to predict poor outcomes |
| 7 | Gong et al. (42) | (D)  Instance weighting | - Weighted Instances: Calculates Euclidean distance of each instance (development and target) from target mean; inverts these distances against the maximum target distance to assign weights. - Weighted Clusters: Uses K-means to cluster development data, then measures Euclidean distance between cluster means and target mean for weighting; closer clusters get higher weights. | Yes | No | - Assumes dataset shift as covariate shift. - Transfers insight from numerous cardiac surgeries. - Development data suitable for clustering in Weighted Clusters method. | The methods perform well in small datasets. | The improved performances were not statistically significant, so the methods are needed to be tested on larger multi-centre datasets. | - Analysing data from 2 hospitals, (2001-11), for cardiac surgery risk stratification.  - Preprocessing this data to include about 250 features, employing (CMIM) to avoid overfitting.  - Fitting models with L2-regularised logistic regression, to address class imbalance - Taking evaluation through LOOCV, measuring performance with precision, recall, and AUC metrics |
| 8 | Lemmon et al. (46) | (D) Predictor selection strategies that enable robustness to temporal dataset shift | Causal feature selection: select features based on causal relationship with the outcome, selected predictors were in Markov Blanket (MB) or Parent-Child (PB)  In addition, there were two proposed methods which we were not include in our review due to insufficient information:  1. L1-regularisation 2. Remove and Retrain (ROAR): identified importance predictors with a permutation-based feature importance score and removed, then retrained the model | Yes | No (except for causal predictor selection) | No explicitly mentioned - it is inferred that the assumption for temporal shift relates to the invariant relationship between outcome and predictor - i.e. covariate shift | Assessing multiple feature selection  methods for transportability across several clinical outcomes | - Only used MIMIC-IV dataset for evaluation. - Inability to predict future predictor shifts. - Lack of a - definitive MB ground truth in MIMIC-IV. - Incomplete assessment of alternative methodologies. | - Analysed ICU patient data from the MIMIC-IV database. - Built logistic regression models trained on 2008-2010 data to predict long LOS, in-hospital mortality, invasive ventilation, and sepsis for 2011-2019. - Evaluation metrics used: AUROC, AUPRC, ACE. |
| 9 | Reps et al. (35) | (D)  Model ensemble | The authors proposed binary ensemble classifiers to increase the generalisability of prediction models. These ensembles are formed by combining individual LASSO logistic regression classifiers, each trained on different databases but predicting the same outcome. The weighted fusion ensemble method assigns weights to these base classifiers, based on their performance metrics or the similarities between the populations they were trained on. It aims to optimise the overall accuracy by effectively utilising the strengths of each classifier. The stacking ensemble method uses predictions from these base learners as input features for a secondary classifier. This secondary classifier adaptively learns the optimal weighting for each base learner's prediction, thereby improving the ensemble's accuracy by tailoring it to the unique characteristics of the dataset being applied to. | No | No | Did not explicitly mention | The study highlights the strength  of the ensemble methods in predicting outcomes for new populations, because of their ability to integrate multiple models trained on diverse datasets. It also was able to compare the transportability of base learners and ensembles in different databases. | The limitation of this study is the generalisability of findings, because the authors studied only for one target population in the US. Miscalibration was also observed for all models. | The study was designed to predict various poor outcomes in patients with pharmaceutically-treated depression, utilising data from both US claims and Electronic Health Records (EHR). The data comprised five datasets, each representing patients with varying characteristics. For model development, base learners were trained using LASSO-penalised logistic regression. To assess the transportability across databases, a leave-one-database-out approach was employed. In this approach, each of the five datasets was excluded in turn to evaluate the performance of the ensemble models. The ensemble models were trained on the remaining four databases and then tested on the excluded database to assess their discrimination and calibration metrics. This process was repeated for each of the five datasets. |
| 10 | de Jong et al. (28) | (D) Predictor selection using  Internal-External Cross-Validation (IECV) of heterogenous training data | 1. Clustering the training dataset to mimic real-world heterogeneity and use ICEV for those clusters to perform stepwise predictor selection 2. From stepwise IECV, aggregate performance metrics of all clusters using meta-analytic methods to select the best model that can generalise across heterogenous dataset | No | 1. The choices of candidate predictors  should be guided by clinical knowledge. | Heterogeneity of training data | - | 1. Predictors are not related to heterogeneity in predictor-outcome associations. 2. Generalisability may come at a cost of improving one predictive performance (such as discrimination), but deteriorating another one. 3. Focusing solely on predictor selection may have an impact on model's precision. 4. It is still possible that the model requires recalibration on the target settings. | The authors demonstrated two case studies: 1. (DVT): Using data from 11 studies (N=10,014), the authors updated a diagnostic model. By applying SIECV to evaluate 10 candidate predictors, they showed that selecting variables based on minimal performance heterogeneity—rather than just average accuracy—led to a more stable model across different clinical settings.  2 (AF): In a prognostic model developed from 20 cohorts (N=10,873), SIECV helped identify predictors that improved overall fit but increased performance variability. This demonstrated that SIECV can prevent overfitting to specific clusters, ensuring more consistent predictions across diverse populations. |
| 11 | Dockès et al. (36) | (D) Importance weighting of training data | Use density ratio weighting to adjust covariate balance between development and target populations | Yes | No | Outcome is independent of Selection given covariates | - | - | - |
| 12 | Pajouheshnia et al. (37) | (D)  Comparability of measurement methods | The discriminative ability of a prediction model varies across settings with variation in the measurement of predictors | Yes | Yes | Assuming normal distribution of predictors | First paper to consider measurement errors as transportability issues | Isolate the effect of predictor measurement from other factors | Case study in DVT prediction to show how increasing measurement errors in different settings affect discrimination performance |
| 13 | Qian et al. (43) | (D)  Adversarial validation | Adversarial validation by mimicking validation data with target population: | Yes | No | - | - | - | Credit score prediction |
| 14 | Subbaswamy et al. (21) | (K) Using causal methods to help transporting a CPM encoded by a graph | Proposing the Graph Surgery Estimator (GSM) by creating a selection diagram having the interventional distribution on the mutable variables and then apply the identifiable algorithm to resolve into the observational distribution | Yes | Yes, domain knowledge requires for both causal relationships  (including unobserved confounders) of each variable in the system, and specification of the variables that vary across environments (mutable variables) | 1. The selection diagram is correctly specified. 2. The interventional distribution is identifiable. |  | 1. Predicting variables is mutable. (prior probability shift) | The authors evaluated the Graph Surgery Estimator against other potential methods through two sets of experiments. In simulated linear Gaussian experiments, the surgery estimator demonstrated superior stability, maintaining a near-constant MSE in various scenarios where both Ordinary Least Squares (OLS) and graph pruning failed. When applied to real-world UCI Bike Sharing data, the surgery estimator achieved the lowest MSE in several test cases and consistently outperforming OLS during high-volatility seasonal shifts. Overall, their findings suggest that graph surgery is more stable than OLS and graph pruning across various dataset shift scenarios. |
| 15 | Pearl et al. (45) | (K)  Formalise transport formula using selection diagram | The statistical relation of interest, such as P(y\|x), can be estimated in the target domain using the following transport formula: P*(y\|x) = ∑c P(y\|x,C) P*(C), where P*(y\|x) represents the transported relation in the target domain, C is a set of covariates that satisfies the condition of S-admissibility. The key aspect is that the marginal probability distribution of C ,P*(C), is the only one that need to be re-estimated in the target domain. | Yes | Yes | 1. Valid selection diagram is required 2. The covariate C need to satisfy S-admissibility, i.e. y ⫫ x \| C |  | - Mainly addresses the causal effect of a single variable X and does not cover prediction scenarios with multiple predictors - Lacks cases where the covariate set C overlaps with the predictor set X, which is common in observational studies - Predominantly remains theoretical | - |
| 16 | Fehr et al. (22) | (K)  Using DAG to help transporting a CPM | Model that takes causes as predictors is more  transportable than those taking consequences | Yes | Yes, to build a DAG | Relies on underlying structural causal equations. | Novel approach to assessing the transportability of CPMs using empirical data and underlying causal structures | DAG needs to be verified. | Using ADNI data to create DAGs and use structural equations to quantify the relationship for the equations, then use the DAGs to test transportability under different situations |
| 17 | Neto et al. (44) | (K)  Counterfactual approach for causality-aware predictions | The methodology aims to adjust confounding in anticausal prediction tasks. The goal is to generate causality-aware predictors that account for confounding variables. Using predictors, confounders and outcomes from training data, the methodology estimates the regression coefficients and residuals and uses this information to create a counterfactual version of predictors that adjusted to remove the influence of confounders. Apply the same process to the test or target data to adjust using the coefficients and residuals from the training set to create the logically-similar counterfactual set of target data. | Yes | Yes | The assumptions need correctly specifying that the predictors are caused by an outcome - anticausal prediction. In addition, working with causal relationship requires domain knowledge. | It does not require full  understanding of causal graph, only the causal relationships of confounders, predictors and outcomes. | The methodology focuses predominately on linear models. It also sensitive to inaccurate specification of variables in the system. | The authors employed linear models with ten  predictors and one confounder, generating samples of training and test sets. They imposed a covariate shift to mimic changes in the joint distribution of confounders and the outcome that resulted from selection biases. The design aimed to reveal that only adjusting confounders in training data is not enough for robust predictions in the face of dataset shifts caused by selection bias. |
| 18 | Bellamy et al. (26) | (K)  Shortcut predictor | A shortcut predictor is any predictor that is associated with an outcome and is site-specific. For example, a watermark in a hospital that specifically admitted COVID19 patients. Identifying and removing shortcut predictors require expert knowledge on causal relationships of them and true predictors. Excluding the shortcut predictor before training a CPM is necessary for it to be generalisable. | Yes | Yes | Correctly specified DAG | - | - | - |
