# Supplementary material for: Scoping review of methodology for aiding generalisability and transportability of clinical prediction models
Source: Diagn Progn Res. 2026 Oct 1;10:25. doi: 10.1186/s41512-026-00237-8 (PMC13629139; doi:10.1186/s41512-026-00237-8)
Supplement: Supplementary file 2 [file 41512_2026_237_MOESM2_ESM.docx]

Appendix 1: formal definitions of the methodology

Density ratio weighting

Using the density ratio for weighted maximum likelihood minimises the Kullback-Leibler divergence between the estimated and true conditional density of the outcome given covariate. This results in the prediction models that are specifically tailored for the target population, which improve the transportability. Mathematically, the density ratio, $r\left( x \right)$ is the ratio of the probability distribution of covariate between target and development population,

$$\begin{aligned} r\left( x \right)=\frac{P_{target}\left( x \right)}{P_{source}\left( x \right)}=\frac{P\left( x | s=0 \right)}{P\left( x | s=1 \right)}\#\left( 1 \right) \end{aligned}$$

where $P_{s}\left( x \right)$ is the probability distribution of covariate $x$ for the population $s$, for which $s=0$ indicates target population and $s=1$ indicates development population. The ratio can be directly calculated using Kernel Mean Matching (KMM) and Relative Unconstrained Least-Squares Importance Fitting (RuLSIF). Furthermore, the ratio can be indirectly estimated by training a classifier to predict the odds of being sampled from the target population,

$$\begin{aligned} \frac{P\left( s=0 | x \right)}{1-P\left( s=0 | x \right)}=\frac{P\left( s=0 | x \right)}{P\left( s=1 | x \right)}\#(2) \end{aligned}$$

By Bayes’ rule, we have:

$$\begin{aligned} P\left( s | x \right)=\frac{P\left( x | s \right)P\left( s \right)}{P\left( x \right)}\#(3) \end{aligned}$$

Therefore:

$$\begin{aligned} \frac{P\left( s=0 | x \right)}{P\left( s=1 | x \right)}=\frac{\frac{P\left( x | s=0 \right)P\left( s=0 \right)}{P\left( x \right)}}{\frac{P\left( x | s=1 \right)P\left( s=1 \right)}{P\left( x \right)}}=\frac{P\left( x | s=0 \right)}{P\left( x | s=1 \right)}\frac{P\left( s=0 \right)}{P\left( s=1 \right)}\#(4) \end{aligned}$$

By rearranging the equation (4), we have the density ratio as estimated from the odds of being sampled from the target population:

$$\begin{aligned} r\left( x \right)=\frac{P\left( s=0 | x \right)}{P\left( s=1 | x \right)}\frac{P\left( s=1 \right)}{P\left( s=0 \right)}\#\left( 5 \right) \end{aligned}$$

where $P(s=0)$ and $P\left( s=1 \right)=1-P(s=0)$ are the probability of being sampled from the target population and the probability of being sampled from the development population, respectively.

$r\left( x \right)$ was described for density ratio weights by Gao et al. (2021) (27) and Steingrimsson et al. (2022) (33). Steingrimsson described this as the inverse of the odds of being from the development population. They showed that the inversed odds is proportional to the inverse odds in the training set,

$$\begin{aligned} \frac{P\left( s=0 | x \right)}{P\left( s=1 | x \right)}\propto\frac{P\left( s=0,x,D_{train}=1 \right)}{P\left( s=1,x,D_{train}=1 \right)},\#\left( 6 \right) \end{aligned}$$

under the assumptions where:

1. the outcome is conditionally independent of the population given the covariates, i.e. no covariate shift, ($P(y|x,s=0) = P(y|x,s=1)$)
2. the probability of covariate patterns that exists in the target population is not zero in the development population, i.e. overlap/positivity, and
3. the study is non-nested design, where the development and target data are separated sampling from different populations – i.e. transportability. By making these assumptions, especially the last assumption, the methodology aid toward improving the transportability of a clinical prediction model.

S-admissibility

S-admissibility is a criterion used to determine if a causal effect measured in a source population can be validly transferred to a target population.

A set of variables $Z$ is S- admissible for the causal effect of $X$ on $Y$ relative to a selection node $S$ if $Y$ is independent of $X$ given $X$ and $Z$.

The probability of a given disease $Y$ (e.g. CVD) in the target population $(S=1)$ can be estimated by standardising the corresponding probability in the development population over a set of covariates that satisfies S-admissibility conditions, assuming the marginal probability of those covariates in the target population is known.

For example:

$$\begin{aligned} P\left( Y=1 | X=x,S=0 \right)=\sum_{Z} P\left( Y=1 | X=x,S=1,Z=z \right)P\left( Z=z \right)\#\left( 6 \right) \end{aligned}$$

In this context, $Y$ is the probability of a disease (CVD), $Z$ is the set of predictors satisfying S-admissibility (statins and smoking), $X$ is a set of other predictors, and $S$ is whether being selected from development ($S=0$) or target ($S=1$) populations.

Appendix 2: Search strategy

Search Date: 4^th^ August 2023

The exact search terms and corresponding results for each databases are shown in the following tables.

Embase

| Search terms | Results |
| --- | --- |
| 1. (generalisab* or generalizab* or transportab* or "dataset shift").ti. | 2322 |
| 2. Validat*.mp or Predict*.ti. or Rule*.mp. or (Predict* and (Outcome* or Risk* or Model*)).mp. or ((History or Variable* or Criteria or Scor* or Characteristic* or Finding* or Factor*) and (Predict* or Model* or Decision* or Identif* or Prognos*)).mp. | 10497047 |
| 3. Stratification OR ROC Curve OR Discrimination OR Discriminate OR c-statistic OR c statistic OR Area under the curve OR AUC OR Calibration OR Indices OR Algorithm OR Multivariable | 2408928 |
| 4. 1 AND (2 OR 3) | 1170 |

MEDLINE

| Search terms | Results |
| --- | --- |
| 1. (generalisab* or generalizab* or transportab* or "dataset shift").ti. | 2087 |
| 2. Validat*.mp or Predict*.ti. or Rule*.mp. or (Predict* and (Outcome* or Risk* or Model*)).mp. or ((History or Variable* or Criteria or Scor* or Characteristic* or Finding* or Factor*) and (Predict* or Model* or Decision* or Identif* or Prognos*)).mp. | 7545662 |
| 3. Stratification OR ROC Curve OR Discrimination OR Discriminate OR c-statistic OR c statistic OR Area under the curve OR AUC OR Calibration OR Indices OR Algorithm OR Multivariable | 1552514 |
| 4. 1 AND (2 OR 3) | 953 |

arXiv

| Search terms | Results |
| --- | --- |
| 1. generalisab or generalizab or transportab or "dataset shift" | 516 |

As Embase and MEDLINE databases were searched through the same Ovid platform, deduplication was done internally. The deduplicated records from the Ovid platform were 1,245 papers. The 1,245 papers (Ovid) and 516 papers (arXiv) were eligible for title screening as per the PRISMA figure (Figure 2 in the main text).
